# Theoretical Derivation of the Effect of Bonding Current on the Bonding Interface during Anodic Bonding of PEG-Based Encapsulation Materials and Aluminum

**DOI:** 10.3390/polym15040913

**Published:** 2023-02-11

**Authors:** Chao Du, Yali Zhao, Yong Li

**Affiliations:** 1 Department of Materials Science and Engineering, Jinzhong University, Jinzhong 030600, China; 2 Shanxi Province Collaborative Innovation Center for Light Materials Modification and Application, Jinzhong 030600, China

**Keywords:** solid polymer electrolyte, anodic bonding, polyethylene glycol, bonding encapsulation, bonding current, interface gap

## Abstract

This study analyzed the mechanism underlying the effect of the bonding current on the bonding interface during anodic bonding on the basis of the anodic bonding of PEG (polyethylene glycol)-based encapsulation materials and Al. By establishing an equivalent electrical model, the effects of various electrical parameters on the dynamic performance of the bonding current were evaluated, and the change law of the bonding current transfer function was analyzed. By examining the gap deformation model, the conditions for contact between the interface gaps and the bonding current pair were determined, and the influence law of the gap deformation of the bonding interface was derived. By assessing the effect of the bonding current on the ionic behavior, we found that the larger the bonding current, the greater the number of activated mobile ions in the bonding material and the higher the field strength in the cation depletion area. From the anodic bonding experiments, it was found that increasing the bonding voltage can increase the peak current and improve the bonding efficiency. The SEM image after bonding shows that the bonding interface had no obvious defects; the higher bonding voltage can result in a thicker bonding layer.

## 1. Introduction

Anodic bonding is an efficient, clean, and reliable process of joining dissimilar materials and has been used to encapsulate various MEMS (Micro-electro Mechanical Systems) devices [1,2,3,4]. In anodic bonding, strong electrostatic and temperature fields are required, the surface properties of the material are altered, and an irreversible physicochemical coupling reaction occurs at the contact point to form a permanent connection, with the role of the bonding current being dominant [5,6,7,8,9,10,11,12,13,14]. Under different bonding conditions (i.e., materials and process parameters), the current variation characteristics during anodic bonding differ. Previous research on anodic bonding has usually focused on the influence of bonding material properties, surface treatment, and bonding parameters on the bonding process, and rarely on the effect of current characteristics on the microscopic changes in bonding materials and the effects of the bonding process [15,16,17,18,19,20,21]. Consequently, the microscopic control and analysis of the entire bonding process are inadequate [22,23,24,25].

In this study, an electrical model of anodic bonding was established and analyzed based on the anodic bonding of a PEG-based solid electrolyte and Al. The effects of the bonding current on the bonding process and bonding quality during anodic bonding were also evaluated. This research provides a theoretical basis for adjusting the bonding process and improving the quality of anodic bonding encapsulation.

## 2. Emergence and Research Methods for the Bonding Current

**Figure 1:**
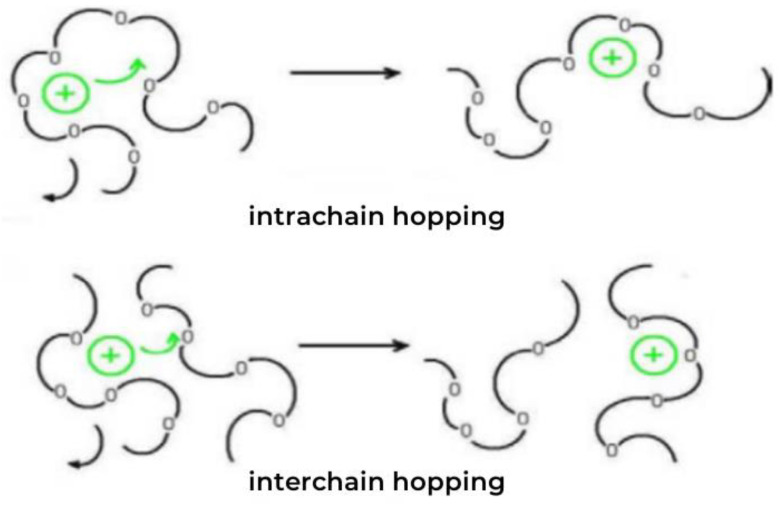
Schematic of Li^+^ migration.

On the basis of the anodic bonding of (PEG)_10_LiClO_4_ and Al, Li^+^ occurring in DSPEs (Dry Solid Polymer Electrolytes) mainly exists in the ionic state [26,27]. During the complexation reaction with PEG, Li^+^ can be dispersed and embedded in the molecular chain of PEG in accordance with certain rules for complexation. At room temperature, owing to the influence of Coulomb force and intermolecular force inside the material, the free movement of Li^+^ is inhibited, and the material exhibits low ionic conductivity. When ambient temperature increases, the thermal motion of the molecules inside DSPE increases, and the energy increases [28,29,30]. When the energy of Li^+^ increases to a certain level, it eliminates the shackles of the surrounding Coulomb force and intermolecular force and then forms a decomplexed state. Li^+^ moves from its original position to another adjacent vacancy, and then moves directionally within the material under an external electric field (Figure 1) [31,32,33,34,35].

Thus, a “decomplexation–complexation–recomplexation” cycle is generated, which can be seen as Li^+^ moves from one molecular chain position to the next vacancy (intrachain hopping). Simultaneously, it moves from one molecular chain to the vacancy on another molecular chain (interchain hopping). This continuous movement, coupled with the action of the electric field, forms a directional current.

By establishing an equivalent electrical model, the electrical parameters affecting the bonding current were identified, and the reasons these parameters influence the current were analyzed.

To explore the mechanism of the bonding current in anodic bonding, we established the bonding interface model, examined the interfacial gap deformation law, and analyzed the role of the bonding current in the gap deformation.

By studying the behavior of interfacial ions during bonding, we aimed to determine the effect of bonding current on the migration of interfacial ions.

## 3. Results and Discussion

### 3.1. Establishment and Analysis of the Electrical Model

Since the current increases rapidly in the initial stage of the bonding process and reaches a peak value within a considerably brief period, to simplify the calculation, we skipped the current growth period and considered the current as the beginning of bonding; that is, when *t* = 0, the peak value is reached (the cation depletion area is formed at this time). An electrical model was thus designed and established.

**Figure 2:**
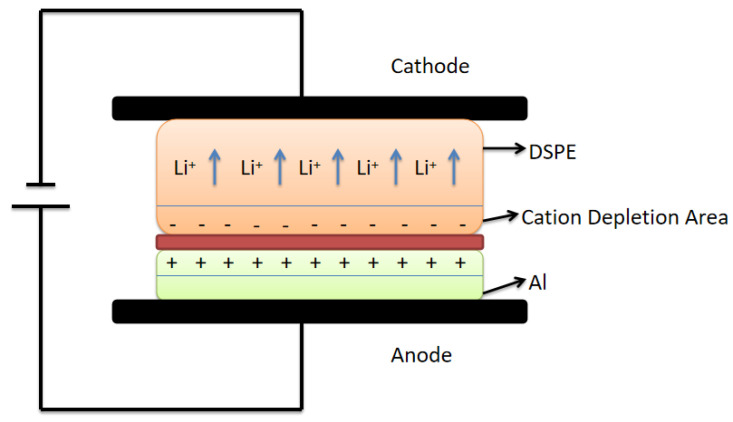
Schematic of ion migration and distribution during bonding.

**Figure 3:**
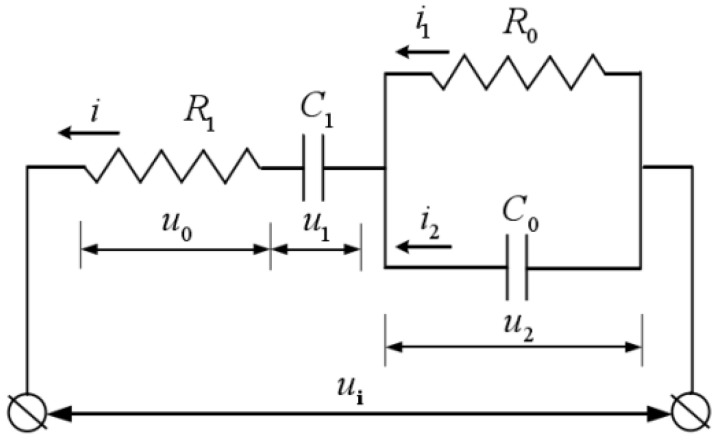
Equivalent electrical model.

Resistance in the circuit was mainly divided into two parts—the resistance *R*_1_ of the electrolyte itself and the resistance *R*_0_ of the contact area between the electrolyte and Al. These areas cannot be in complete contact with each other; small gaps are present in uncontacted areas. This void is equivalent to capacitance *C*_0_. As bonding progresses, a cation depletion area occurs (Figure 2) inside the polymer near the bonding interface. This area is equivalent to capacitance *C*_1_, hence the presence of electrolyte resistance *R*_1_, the cation depletion area *C*_1_, material contact part resistance *R*_0_, and the noncontact part *C*_0_, with *R*_0_ and *C*_0_ being in a parallel relationship (Figure 3).

We assume that the voltage across *R*_1_ is $u_{0}\left(t\right)$; thus, the total current in the circuit i(t)

(1) $$i\left(t\right)=\frac{u_{0}\left(t\right)}{R_{1}}$$

The voltage across *C*_1_ is given by

(2) $$u_{1}\left(t\right)=\frac{1}{C_{1}}\int_{0}^{t}i\left(t\right)\mathrm{dt}=\frac{1}{R_{1}C_{1}}\int_{0}^{t}u_{0}\left(t\right)\mathrm{dt}$$

The voltage across the parallel part of *R*_0_ and *C*_0_ and the current on each branch are $u_{2}\left(t\right)$,$\;i_{1}\left(t\right)$, and $i_{2}\left(t\right)$, respectively, and are calculated as follows:

(3) $$u_{2}\left(t\right)=u_{i}\left(t\right)-u_{0}\left(t\right)-\frac{1}{R_{1}C_{1}}\int_{0}^{t}u_{0}\left(t\right)\mathrm{dt}$$

(4) $$i_{1}\left(t\right)=\frac{1}{R_{0}}[u_{i}\left(t\right)-u_{0}\left(t\right)-\frac{1}{R_{1}C_{1}}\int_{0}^{t}u_{0}\left(t\right)\mathrm{dt}$$

(5) $$i_{2}\left(t\right)=C_{0}\frac{du_{2}\left(t\right)}{\mathrm{dt}}=C_{0}\left[\frac{du_{i}\left(t\right)}{\mathrm{dt}}-\frac{du_{0}\left(t\right)}{\mathrm{dt}}-\frac{1}{R_{1}C_{1}}u_{0}\left(t\right)\right]$$

The total current in the circuit is expressed as i(t)=$i_{1}\left(t\right)$+$i_{2}\left(t\right)$. The function model (transfer function) in the frequency domain can be obtained using the Laplace transform. We assume that at *t* = 0, *u_i_*, and *u*_0_ and their derivatives are 0, and the closed-loop transfer function is obtained as follows:

(6) $$\Phi \left(s\right)=\frac{u_{0}\left(s\right)}{u_{i}\left(s\right)}=\frac{s^{2}+\frac{1}{R_{0}C_{0}}s}{s^{2}+\left(\frac{1}{R_{0}C_{0}}+\frac{1}{R_{1}C_{1}}+\frac{1}{R_{1}C_{0}}\right)s+\frac{1}{R_{0}C_{0}R_{1}C_{1}}}$$

Let $\omega_{n}=\sqrt{\frac{1}{R_{0}C_{0}R_{1}C_{1}}}$,$\;\zeta =\frac{R_{0}C_{0}+R_{1}C_{1}+R_{0}C_{1}}{2\sqrt{R_{0}C_{0}R_{1}C_{1}}}$,$\;K=\frac{1}{R_{0}C_{0}}$. Via substitution into the aforementioned formula, the following is derived:

(7) $$\Phi \left(s\right)=\frac{s^{2}+Ks}{s^{2}+2\zeta \omega_{n}s+\omega_{n}^{2}}$$

Since $R_{0}C_{0}+R_{1}C_{1}\geq 2\sqrt{R_{0}C_{0}R_{1}C_{1}}$, substitution into (7) yields

(8) $$\zeta =\frac{R_{0}C_{0}+R_{1}C_{1}+R_{0}C_{1}}{2\sqrt{R_{0}C_{0}R_{1}C_{1}}}>1$$

That is, the equivalent electrical model is an overdamped second-order system. We assume that its input and output responses exert the effect of various electrical parameters on the bonding current. The input to the system is assumed to be a unit step input,$\;U_{i}\left(s\right)=1/s$; thus, the system output response is

(9) $$U_{0}\left(s\right)=\Phi \left(s\right)U_{i}\left(s\right)=\frac{s+K}{s^{2}+2\zeta \omega_{n}s+\omega_{n}^{2}}$$

The current unit step response in the circuit is given by

(10) $$I\left(s\right)=\frac{U_{0}\left(s\right)}{R_{1}}=\frac{1}{R_{1}}\frac{s+K}{s^{2}+2\zeta \omega_{n}s+\omega_{n}^{2}}$$

As shown in (10), the unit step response of the bonding current in the circuit is mainly determined in accordance with the four parameters *R*_0_, *C*_0_, *R*_1_, and *C*_1_. In accordance with the formula and the characteristics of the two bonding materials, we can conclude that when *R*_1_ and *C*_1_ are constant, the changes in *R*_0_ and *C*_0_ do not change the peak current during bonding. When *R*_0_ and *C*_0_ are constant, the smaller the value of *R*_1_ and the larger the value of *C*_1_, the higher the bonding peak current.

### 3.2. Influence of the Bonding Process on Electrical Parameters

#### 3.2.1. Bonding Temperature

The effect of the bonding temperature on electrical parameters is mainly reflected in the effect of temperature on the ionic conductivity of electrolyte materials. This is because at room temperature, the transportable ions in the solid polymer electrolyte are constrained by the Coulomb force and the surrounding intermolecular force, and it is difficult to move freely. This not only affects the bulk resistance of the material, but also limits the number of effective carrier migrations during the bonding process, which then affects the current change during the bonding process [17,18,19]. When the bonding temperature is increased, the internal structure of the solid polymer electrolyte is activated, thus generating higher molecular thermal motion energy and realizing the free movement of ions [36,37,38]. This is similar to conclusions drawn in previous research. The ionic conductivity is increased, which affects the bonding current. That is, if the bonding temperature is increased, the reaction to the electrical parameters is the decrease in *R*_1_. However, the bonding temperature may not be increased to reduce the bulk resistance blindly because the increase in temperature can cause the material to soften, preventing bonding [39,40].

In our study, we selected different bonding temperatures, from room temperature to 50 °C. After measurement, the ionic conductivity of the material was calculated based on the above principles. Materials in this temperature range can be bonded; that is, the strength of the material will not be greatly reduced due to excessive softening caused by high temperature.

#### 3.2.2. Bonding Electrode

**Figure 4:**
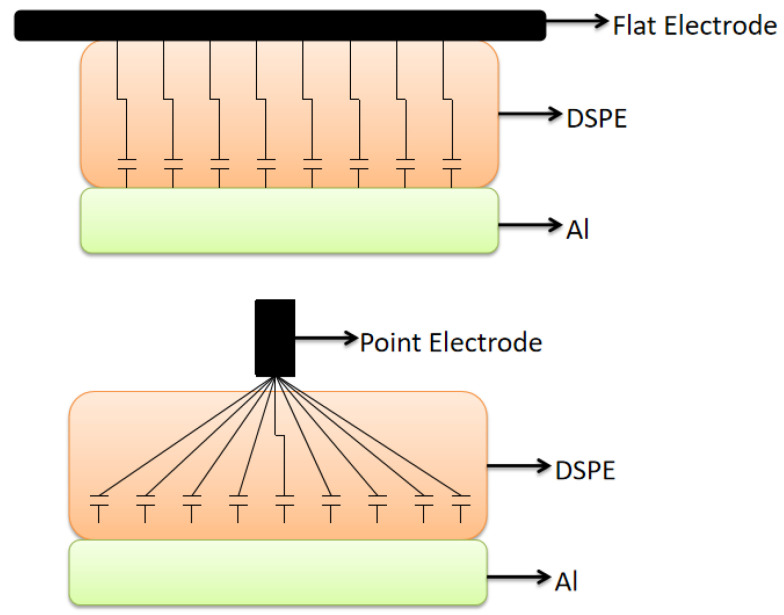
Equivalent parallel circuit schematic under plate and spot electrodes.

The most commonly used bonding electrodes are flat and point electrodes (Figure 4). The resistance of the material is equivalent to the resistance of the branches formed from each point in the material to the parallel electrode, and the two electrodes vary greatly in length from each point in the material to the electrode. The greater the length, the higher the resistance.

When using a flat electrode, the length from each point in the material to the electrode is equal to the thickness of the material, and the overall resistance of the material is low after a parallel connection. When using a point electrode, except for the points in the material perpendicular to the electrode, the remaining points are connected to the electrode. The length of the electrode is greater when using a point electrode than when using a flat electrode. Under certain conditions, the resistance of the material using the flat electrode is lower, and the peak current generated during the bonding process is larger.

### 3.3. Analysis of the Bonding Interface

#### 3.3.1. Construction of the Bonding Interface Model

**Figure 5:**
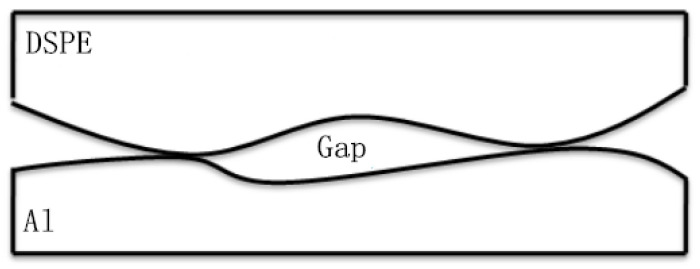
Diagram of the interface contact.

In the anodic bonding of DSPEs (Dry Solid Polymer Electrolytes) and metals, the materials to be joined need to be in close contact to facilitate bonding. However, a tight fit requires material deformation. Actual bonding requires numerous point contacts; thus, many microscopic gaps are found at the bonding interface (Figure 5).

**Figure 6:**
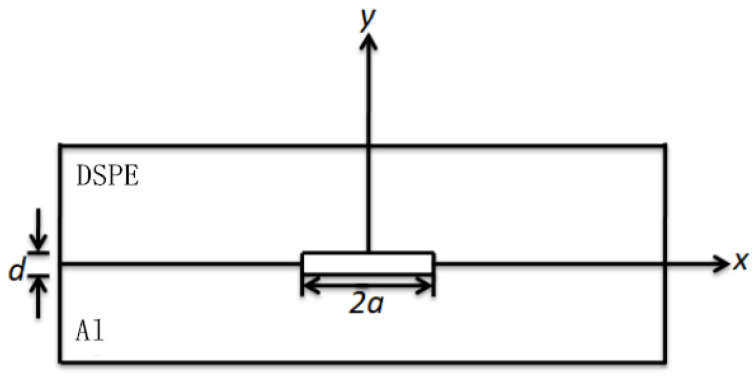
Bonding interface model.

To facilitate the analysis and calculation of gap deformation, we approximately simplified the actual interface to a plane interface model (Figure 6). The interface gap width is set to 2a, and the depth is d, under the assumption that both the width and depth are smaller than the material thickness, and the depth is much smaller than the width. The z-axis direction was not considered, hence the conversion of bond material interface gap deformation into plane strain for continued analysis.

#### 3.3.2. Analysis of Interfacial Gap Deformation

In Figure 6, we assume that neither shear force at the well-contacted bonding interface (|x| > *a*) nor displacement in the y-axis exists. The maximum deformation of the gap should be at the origin of the coordinates. We also assume that the elastic modulus of the aluminum foil is $E_{\mathrm{Al}}$, the Poisson’s ratio is *v*_Al_, and the pressure on the upper and lower surfaces of the interface gap is P. The deformation of the aluminum foil in the y-axis direction at the gap is thus defined as

(11) $$u_{\mathrm{Al}}=2\left(1-v_{\mathrm{Al}}^{2}\right)\frac{P}{E_{\mathrm{Al}}}{\left(a^{2}-x^{2}\right)}^{\frac{1}{2}}$$

The solid polymer electrolyte can be regarded as a linear viscoelastic material in the bonding environment. Assuming that the elastic modulus is E_DSPE_, the Poisson’s ratio is *v*_DSPE_, and the viscosity is η, the creep equation during bonding can be expressed as

(12) $$C\left(t\right)=\frac{1}{E_{\mathrm{DSPE}}}+\frac{t}{\eta }$$

When |x| < *a*, the deformation of the solid polymer electrolyte in the y-axis direction is

(13) $$u_{\mathrm{DSPE}}=2\left(1-v_{\mathrm{DSPE}}^{2}\right){\left(a^{2}-x^{2}\right)}^{\frac{1}{2}}P\int_{0^{-}}^{t}C\left(t-\tau \right)d\tau$$

We then assume that the bonding process is an ideal process in which the current increases and then decreases to zero. That is, when the bonding ends (*t* > *t*_end_), the bonding current is zero, and the voltage is also zero; moreover, the bonding current and voltage always exceed zero during the bonding process. Two relationships can thus be obtained during and after bonding:

(14) $$u_{\mathrm{DSPE}}=2\left(1-v_{\mathrm{DSPE}}^{2}\right){\left(a^{2}-x^{2}\right)}^{\frac{1}{2}}\mathrm{PC}\left(t\right),t\leq t_{\mathrm{end}}$$

(15) $$u_{\mathrm{DSPE}}=2\left(1-v_{\mathrm{DSPE}}^{2}\right){\left(a^{2}-x^{2}\right)}^{\frac{1}{2}}P\left[C\left(t\right)-C\left(t-t_{\mathrm{end}}\right)\right],t>t_{\mathrm{end}}$$

Owing to the low-temperature bonding process used in this project, the variation in bonding temperature is considerably small. At temperatures ranging from 50 °C to 80 °C, the viscosity of PEG-based bonding material is about 10^20^ Pa·s, and its elastic modulus is E_DSPE_ = 1200 MPa. Ignoring creep due to viscous flow, we derive the total deformation:

(16) $$u=u_{\mathrm{DSPE}}+u_{\mathrm{Al}}=2P{\left(a^{2}-x^{2}\right)}^{\frac{1}{2}}\left(\frac{1-v_{\mathrm{Al}}^{2}}{E_{\mathrm{Al}}}+\frac{1-v_{\mathrm{DSPE}}^{2}}{E_{\mathrm{DSPE}}}\right)$$

The maximum deformation at the origin of the coordinates is

(17) $$u_{max}=2\left(1-v_{\mathrm{Al}}^{2}\right)\frac{P}{E_{\mathrm{Al}}}a+2\left(1-v_{\mathrm{DSPE}}^{2}\right)\frac{P}{E_{\mathrm{DSPE}}}a=2\frac{P}{E_{\mathrm{eff}}}a$$

(18) $$\frac{1}{E_{\mathrm{eff}}}=\frac{1-v_{\mathrm{DSPE}}^{2}}{E_{\mathrm{DSPE}}}+\frac{1-v_{\mathrm{Al}}^{2}}{E_{\mathrm{Al}}}$$

where $E_{\mathrm{eff}}$ is the effective Young’s modulus. In the anodic bonding experiment, if the solid electrolyte is closely attached to the aluminum foil, the total deformation of the gap is greater than or equal to the depth of the gap, denoted as $u_{max}\geq d$. Then, $P\geq \frac{E_{\mathrm{eff}}\cdot d}{2a}$; that is, the electrostatic voltage is not less than $\frac{E_{\mathrm{eff}}\cdot d}{2a}$.

Gap deformation is related to gap pressure, which is related to gap voltage. For convenience in research and calculation, we assume that the electrostatic field environment field strength is uniform, the voltage at the interface gap is *V_gap_*, the field strength is *E_gap_*, and *V_gap = _E_gap_*·*d*. The strength of the gap electric field is formed between the upper and lower surfaces of the two materials at the interface gap; that is, the effect of a single surface cannot be ignored. The field strength *E* generated by one of the surfaces can be approximately calculated as:

(19) $$E=\frac{E_{gap}}{2}=\frac{V_{gap}}{2d}$$

(20) $$\sigma_{s}=\frac{Q}{S}=\frac{CV_{gap}}{S}$$

where $\sigma_{s}$ is the surface charge density near the aluminum foil at the interfacial gap, Q is the gap charge, *S* is the surface area, and *C* is the gap capacitance. In accordance with the definition of capacitance, combined with (20),$\;\sigma_{s}=\frac{\varepsilon_{0}\varepsilon_{r}V_{gap}}{d}\approx \frac{\varepsilon_{0}V_{gap}}{d}$, where *ε*_r_ is the relative permittivity and can be approximately considered equal to 1, and *ε*_0_ is the vacuum permittivity and is equal to 8.85 × 10^−12^ F·m^−1^. Thus, the electrostatic force on the gap surface is P ($P=E\cdot \sigma_{s}=\frac{\varepsilon_{0}V_{gap}^{2}}{2d^{2}}$). Comprehensive analysis shows that the gap voltage has to satisfy the following:

(21) $$V_{gap}>{\left(\frac{E_{\mathrm{eff}}d^{3}}{\varepsilon_{0}a}\right)}^{\frac{1}{2}}$$

Therefore, under the given bonding material and anodic bonding parameters, the gap voltage is an important factor in determining the interface gap deformation. When the gap voltage exceeds ${\left(E_{\mathrm{eff}}d^{3}/\varepsilon_{0}a\right)}^{1/2}$, the amount of interface deformation is optimal, and the two bonding materials exhibit the best fit. This conclusion has not been clarified in previous inferences.

#### 3.3.3. Effect of the Bonding Current on Interfacial Gap Deformation

Based on previous analysis, the relationship between gap voltage and interface gap deformation is drawn; the gap voltage is related to the gap current. The current is analyzed using Figure 3. The aforementioned analysis, combined with Kirchhoff’s voltage law analysis, results in the voltage *C*_1_:

(22) $$u_{1}\left(t\right)=u_{i}\left(t\right)-R_{1}\left[C_{0}\frac{du_{2}\left(t\right)}{dt}+\frac{u_{2}\left(t\right)}{R_{0}}\right]-u_{2}\left(t\right)$$

By using the capacitor *C*_1_, the main circuit current can be expressed as

(23) $$i\left(t\right)=C_{1}\frac{du_{1}\left(t\right)}{dt}=C_{1}\left[\frac{du_{i}\left(t\right)}{dt}-\left(1+\frac{R_{1}}{R_{0}}\right)\frac{du_{2}\left(t\right)}{dt}-R_{1}C_{0}\frac{d^{2}u_{2}\left(t\right)}{dt^{2}}\right]$$

Moreover,

(24) $$\frac{d^{2}u_{2}\left(t\right)}{dt^{2}}+\left[\frac{1}{R_{0}C_{0}}+\frac{1}{R_{1}C_{0}}+\frac{1}{R_{1}C_{1}}\right]\frac{du_{2}\left(t\right)}{dt}+\frac{u_{2}\left(t\right)}{R_{0}R_{1}C_{0}C_{1}}=\frac{1}{R_{1}C_{0}}\frac{du_{i}\left(t\right)}{dt}$$

We assume that at *t* = 0, $u_{i}$, and $u_{2}$ and their derivatives are zero. The Laplace transform of (24) can thus be obtained:

(25) $$\frac{u_{2}\left(s\right)}{u_{i}\left(s\right)}=\frac{\frac{1}{R_{1}C_{0}}s}{s^{2}+\left(\frac{1}{R_{0}C_{0}}+\frac{1}{R_{1}C_{0}}+\frac{1}{R_{1}C_{1}}\right)s+\frac{1}{R_{0}R_{1}C_{0}C_{1}}}$$

From previous research, we know that the current unit step response I(s) and the input voltage $U_{i}\left(s\right)$ should satisfy the following:

(26) $$\frac{I\left(s\right)}{U_{i}\left(s\right)}=\frac{1}{R_{1}}\frac{s^{2}+\frac{1}{R_{0}C_{0}}s}{s^{2}+\left(\frac{1}{R_{0}C_{0}}+\frac{1}{R_{1}C_{0}}+\frac{1}{R_{1}C_{1}}\right)s+\frac{1}{R_{0}R_{1}C_{0}C_{1}}}$$

By (25) and (26):

(27) $$\frac{U_{2}\left(s\right)}{I\left(s\right)}=\frac{1}{C_{0}s+\frac{1}{R_{0}}}$$

We assume that *b* is the initial peak of the current, *T* is the current half-life (indicating the decay rate), and$\;i\left(t\right)=b{\left(\frac{1}{2}\right)}^{\frac{t}{T}}$ is the bonding current. By further transforming it, the current unit step response is drawn:

(28) $$I\left(s\right)=\frac{b}{s+\left(ln2\right)/T}$$

By substituting (28) into (27), the gap voltage is thus derived:

(29) $$V_{gap}=u_{2}\left(s\right)=\frac{b/C_{0}}{\left(s+\frac{ln2}{T}\right)\left(s+\frac{1}{R_{0}C_{0}}\right)}$$

Therefore, under a given condition, the greater the bonding current, the higher the gap voltage. The gap deformation is therefore increased, and the bonding interface gap is reduced; the upper and lower surfaces of the bonding material can be attached closely. It also facilitates bonding. *V_gap_* is an important factor in determining the deformation of the interface gap. The relationship between *V_gap_* and the bonding current can be determined, and the bonding current can be increased based on the adjustment of the electrical parameters to adjust *V_gap_* and reduce the interface gap. The bonding efficiency is ultimately improved.

### 3.4. Analysis of the Bonding Current and Interfacial Ion Behavior

The influence of the bonding current on the ionic behavior of the bonding interface is manifested in the number of mobile ions in the catholyte material and the field strength in the cation depletion area. PEG-based solid electrolyte materials exhibit very low conductivity, and the internal mobile ions are few. Under the action of the temperature and electrostatic fields during bonding, the ions in the material become “activated”. This occurrence is exacerbated as the bonding current increases (to a certain extent).

**Figure 7:**
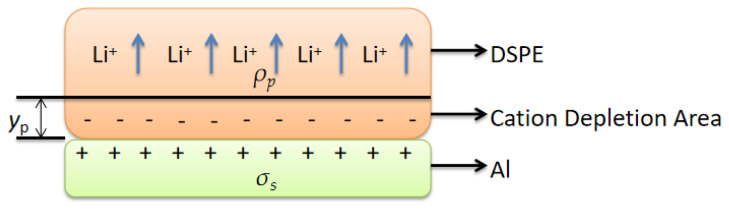
Interface ion distribution map.

To facilitate the assessment of the effect of the bonding current on the internal field strength of the cation depletion area, we assume that the cations can be precipitated after migrating to the cathode, and the oxygen anions in the cation depletion area are uniformly distributed. We further assume that the thickness of the cation depletion area is *y_p_*, the charge density is *ρ_p_*, the dielectric constant is *ε_p_*, and the surface charge density of the aluminum foil is *σ_s_* (Figure 7).

The thickness of the cation depletion area is considerably limited. Regardless, we regard it as an approximately one-dimensional problem. Assuming that the potential from the cation depletion area to the bonding surface y is A, its one-dimensional Poisson equation is expressed as

(30) $$\frac{d^{2}\phi \left(y\right)}{dy^{2}}=-\frac{\rho_{p}}{\varepsilon_{p}}$$

After two integrals:

(31) $$\phi \left(y\right)=-\frac{\rho_{p}}{2\varepsilon_{p}}y^{2}+Ay+B$$

where A and B are constants. The point on the bonding surface, that is, *y* = 0, yields the following:

(32) $$\frac{d\phi \left(0\right)}{dy}=\frac{\sigma_{s}}{\varepsilon_{p}}=\frac{\rho_{p}y_{p}}{\varepsilon_{p}}$$

(33) $$\phi \left(0\right)=\phi_{a}$$

where $\;\;\phi_{a\;}\;$ is the potential at the bonding interface. Thus, the potential at any point in the cation depletion area is

(34) $$\phi \left(y\right)=-\frac{\rho_{p}}{2\varepsilon_{p}}y^{2}+\frac{\rho_{p}y_{p}}{\varepsilon_{p}}+\phi_{a}$$

We assume that *y*_p_ is the thickness of the depletion area, *ε*_g_ is the dielectric constant of the depletion area, and *y* is the distance from a point in the cation depletion area to the interface. The internal field strength of the cation depletion area can thus be calculated as

(35) $$E\left(y\right)=\frac{\partial \phi }{\partial y}=\frac{\rho_{p}}{\varepsilon_{g}}\left(y_{p}-y\right)$$

Current generation during bonding occurs via the directional migration of freely mobile ions in the material, the migrating ions are mainly lithium ions, and the bonding current is integrated to derive the following:

(36) $$Q\left(t\right)=\int_{0}^{t}i\left(t\right)dt=\rho_{p}Sy_{p}e$$

where *S* is the contact area between the solid electrolyte and Al, e is the charge, and *i*(*t*) is the current. The thickness of the ion to the depletion area is thus given by

(37) $$y_{p}=\frac{1}{S\rho_{p}e}\int_{0}^{t}i\left(t\right)dt$$

From (35) and (37), the internal field strength of the cation depletion area can be calculated as

(38) $$E\left(y\right)=\frac{\rho_{p}}{\varepsilon_{g}}\left[\frac{1}{S\rho_{p}e}\int_{0}^{t}i\left(t\right)dt-y\right]$$

As deduced from the formula, when the bonding current increases during bonding, the field strength inside the cation depletion area can also increase. The number of oxygen anions increases; similarly, the number of effective ion migration also increases. Therefore, increasing the current within a certain range during bonding contributes to the bonding efficiency and quality of the material.

### 3.5. Anodic Bonding of PEG-LiClO_4_ and Al

#### 3.5.1. Analysis of Time–Current

**Figure 8:**
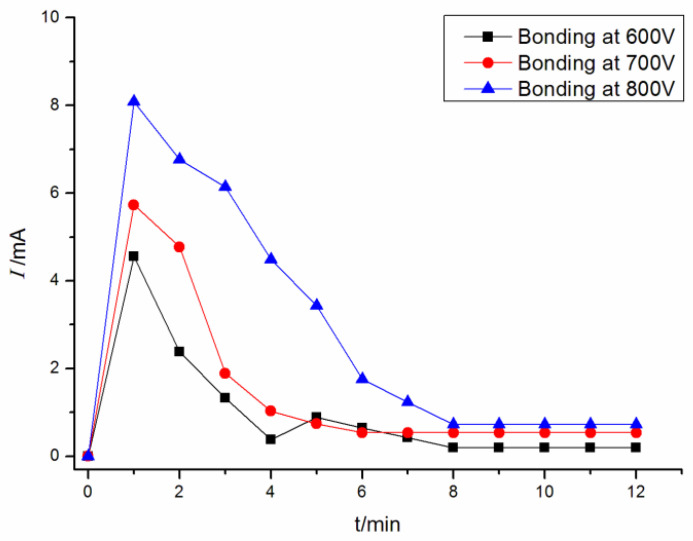
The time–current curve during anode bonding of (PEG)_10_LiClO_4_ and Al.

Figure 8 presents the time–current curve of (PEG)_10_LiClO_4_ and Al in anodic bonding at different bonding voltages. It can be seen that the peak current of bonding for (PEG)_10_LiClO_4_ and Al was 4.55 mA at 600 V, and the peak current increased to 5.73 mA and 8.08 mA at 700 V and 800 V, respectively. This shows that the peak current increased with the increase in bonding voltage.

**Figure 9:**
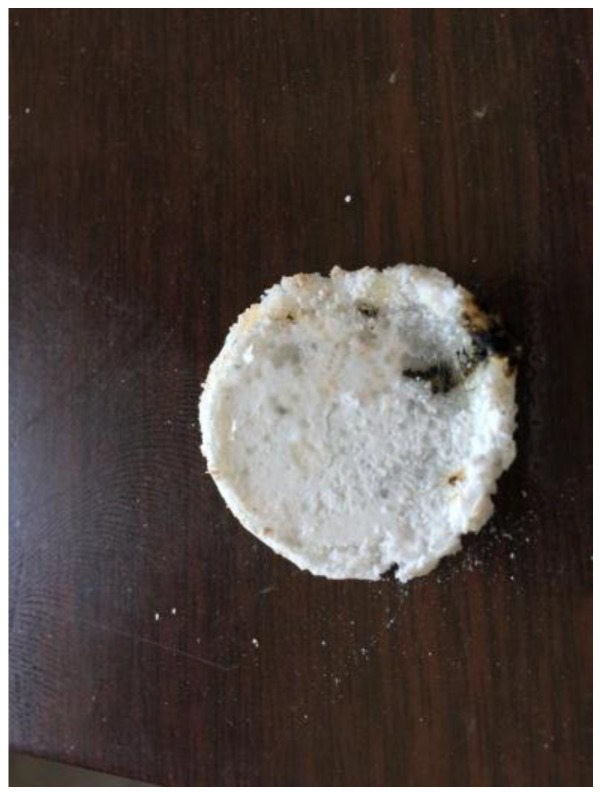
The image of (PEG)_10_LiClO_4_ after breakdown.

The higher bonding voltage can promote the bonding interface to be closely attached, promote ion migration and improve the bonding efficiency. However, further increasing the bonding voltage may break down the bonding material and cause the bonding to fail. Figure 9 shows the bonding interface with the bonding voltage at 850 V. It can be seen that part of the solid polymer electrolyte broke down (the black part), and the bonding failed.

#### 3.5.2. Analysis of Bonding Interface

**Figure 10:**
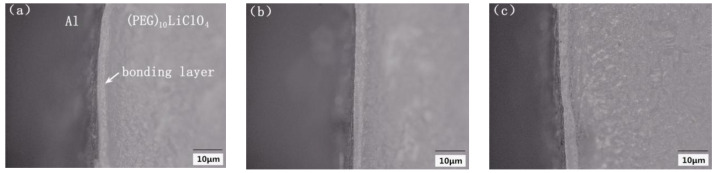
The SEM image of bonding interface of (PEG)_10_LiClO_4_ and Al: (**a**) bonding at 600 V, (**b**) bonding at 700 V, (**c**) bonding at 800 V.

Figure 10 is the SEM (Scanning Electron Microscope) image of the anodic bonding interface of (PEG)_10_LiClO_4_ and Al at different bonding voltages. It can be seen that each interface has an obvious bonding layer between (PEG)_10_LiClO_4_ and Al and there are no obvious pores and cracks at the interface. However, it can be observed that the thickness of the bonding layer increases with the increase in bonding voltage.

In order to more intuitively see the influence of different voltages on the bonding quality, we carried out tensile tests on the bonded interface. The mechanical properties of the bonding interface were studied. The tensile strength of the bonding interface increases with the increase in bonding voltage (Table 1). However, a high bonding voltage will lead to bonding failure.

**Figure 11:**
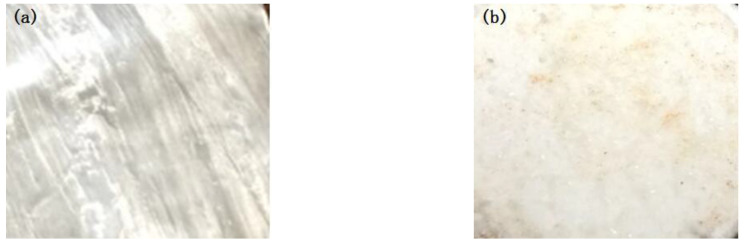
The tensile fracture morphology of sample 3-1: ((**a**) aluminum foil; (**b**) DSPE).

It can be seen from Figure 11 that there is a thin layer of residual material on the surface of one side of the aluminum foil, indicating that its fracture occurred in the bonding layer. However, the interface strength was not ideal at this time (0.6 MPa).

**Figure 12:**
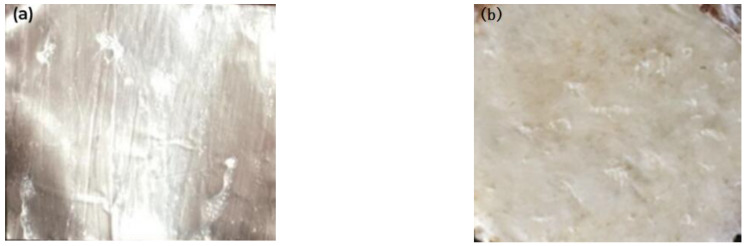
The tensile fracture morphology of sample 3-2 ((**a**) aluminum foil; (**b**) DSPE).

A layer of residue can be seen in Figure 12, and some wire-like residue can also be seen on the surface of (PEG) _10_LiClO_4_. These are all part of the bonding layer, indicating that its fracture occurred in the bonding layer. At this time, the intensity was improved.

**Figure 13:**
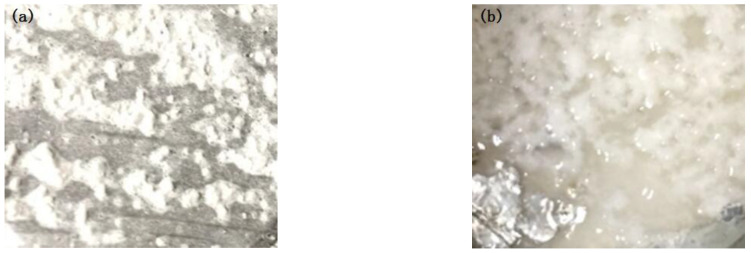
The tensile fracture morphology of sample 3-3: ((**a**) aluminum foil; (**b**) DSPE).

In Figure 13, there significant residue can be seen on one side of the aluminum foil, as well as some pits and a small amount of aluminum foil residue on the polymer side. This shows that part of the tensile fracture of the interface occurred in the bonding base metal and the bonding strength was high at this time (1.3 MPa).

To a certain extent, a high bonding voltage can tighten the interface and improve the carrier concentration. The peak current in bonding also increases. A higher bonding voltage can improve the efficiency of the bonding reaction, increase the thickness of the bonding layer, and improve the bonding quality. However, when the bonding voltage is too high, the bonding interface has almost no strength because of the bonding failure.

## 4. Conclusions

In this study, the effect of the bonding current on the bonding interface was analyzed from the theoretical derivation, which bears certain significance for enhancing the efficiency of anodic bonding encapsulation and improving the connection quality. More importantly, this is the first time theoretical analysis has been conducted on the current of the anodic bonding between PEG-based polymer solid electrolyte and metal. Specific conclusions are thus presented as follows:

By establishing an equivalent electrical model, the electrical parameters affecting the bonding current were identified. Through the analysis of the bonding interface gap model, the conditions under which the bonding interface gaps can contact each other during bonding were derived: gap static voltage strength, P ≥ $E_{\mathrm{eff}}\cdot d/2a$, and gap voltage, *V*_gap_ > ${\left(E_{\mathrm{eff}}d^{3}/\varepsilon_{0}a\right)}^{1/2}$. The influence of the bonding current on interface deformation was also determined: the greater the bonding current, the higher the gap voltage, the larger the interface gap deformation, and the tighter the bonding material. By assessing the effect of the bonding current on ionic behavior, the effects of the bonding current on the number of mobile ions, electric field strength, and ion migration are evaluated. The higher the internal field strength, the greater the number of negative oxygen ions and lithium ions as the main carriers, the more effective the ion migration, and the more appropriate the bonding reaction at the bonding interface.

## Figures and Tables

**Table 1:** The tensile strength of bonding interface between (PEG)_10_LiClO_4_ and Al at room temperature.

Sample	Bonding Voltage (V)	Thickness of (PEG)_10_LiClO_4_ (cm)	Area of Bonding Interface (cm^2^)	Tensile Strength (MPa)
3-1	600	0.31	4.9	0.6
3-2	700	0.32	4.9	0.9
3-3	800	0.32	4.9	1.3
3-4	850	0.32	4.9	—

## Data Availability

The data presented in this study are available on request from the corresponding author.
